# Effect of curcumin on the quality properties of millet fresh noodle and its inhibitory mechanism against the isolated spoilage bacteria

**DOI:** 10.1002/fsn3.1427

**Published:** 2020-02-08

**Authors:** Ziyuan Wang, Yating Jia, Min Zhang

**Affiliations:** ^1^ Beijing Advanced Innovation Center for Food Nutrition and Human Health Beijing Technology and Business University Beijing China; ^2^ Beijing Engineering and Technology Research Center of Food Additives Beijing Technology and Business University Beijing China

**Keywords:** curcumin, inhibitory mechanism, millet fresh noodle, quality

## Abstract

Fresh noodle product has attracted increasing attention due to its nutritive value and convenience. However, the relative short shelf life of fresh noodle is still a concern that needs to resolve. The objective of this study was to evaluate the preservative effect of curcumin (CUR) on millet fresh noodle during storage and its inhibitory mechanism against two isolated spoilage bacteria (*Bacillus cereus* and *Escherichia coli*). The effects of CUR were evaluated with regard to the quality and sensory evaluation of millet fresh noodle, the changes of bacterial growth curve, cell intracellular substances, cell viability, and bacterial morphology. The results showed that CUR could decrease the total colony number and prolong the shelf life of millet fresh noodle stored at 25°C from 20 to 30 hr. Quality and sensory evaluations showed that addition of CUR caused no negative effect on noodle quality and was determined to be sensory acceptable. The minimum inhibitory concentration of CUR against *B.* *cereus* and *E. coli* was 0.125 and 0.5 mg/ml, respectively. The growth curve revealed that CUR presented good antibacterial effect against both bacteria. The leakage of intracellular substances, cell viability, and bacterial morphology change after CUR treatment confirmed the destructive effects of CUR on plasma membrane integrity. These results indicated that CUR had the potential to be applied as a natural preservative for controlling the growth of spoilage microorganisms and extending the shelf life of millet fresh noodle.

## Graphical abstract

Curcumin was able to prolong the product's shelf life to 30 hr when stored at 25°C without causing decline in sensory property. Curcumin inhibited the growth of two spoilage bacteria (*Bacillus cereus* and *Escherichia coli*) isolated from fresh millet noodle. Curcumin could cause cell intracellular substances leakage and changes of bacterial morphology and plasma membrane integrity.

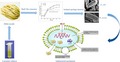

## 1 INTRODUCTION

With the improvement of living standard, consumers have increased demand for high quality and convenient foods such as instant, refrigerated, and frozen foods. Nowadays, various noodle products have been growing due to the convenience and affordability. The common types of noodle include dried, fried, instant, frozen, and fresh noodle. Especially for fresh noodle, they are increasingly popular and becoming the competing product with dried noodle that has been dominated the market for a long time (Lee, Kang, & Kim, 2017). The main advantages of fresh noodle are their high nutritional value, good palatability, and easy preparation. In general, the majority of noodle are made from wheat flour and the addition of novel substitutes which can increase variety and functionality to noodle products has caused more attention (Liu et al., 2018; Wang et al., 2016). For instance, many noodle products made from composite flours have been reported, including sweet potato flour (Chen, Schols, & Voragen, 2003), taro flour (Srikaeo, Mingyai, & Sopade, 2011), and cassava (Charles et al., 2007).

Currently, one of the main concerns for fresh noodle products is the short shelf life, due to its high moisture content (~35%) and water activity (Aw > 0.96) which could lead to food contamination by spoilage bacteria (Hou, Li, Wang, Sun, & Mo, 2017). As high temperature treatment would destroy the structure and texture of starch‐based products, it is not suitable to use heat‐related sterilization for fresh noodle products. In this case, using natural antimicrobial compounds without causing negative impact on food quality (e.g., color, texture, and sensory acceptability) turns into a practical way for quality control and becomes a new trend in food production (Fei et al., 2018; Fei, Xu, Zhao, Gong, & Guo, 2019). Curcumin originating from Curcuma longa rhizome or turmeric is known to possess diverse biological and pharmacological activities, such as anti‐inflammatory (Verma et al., 2019), antioxidant (Asouri, Ataee, Ahmadi, Amini, & Moshaei, 2013), antibacterial (Sathishkumar et al., 2015), and antimutagenic (Anto, George, Babu, Rajasekharan, & Kuttan, 1996). In addition, curcumin has good clinical safety with relative nontoxicity and has a long history of use as herbal remedy, dietary spice, and food‐coloring agent in East Asia (Lao et al., 2006; Shoba et al., 1998).

*Bacillus cereus and Escherichia coli* are the two major bacteria isolated from fresh noodles made in our laboratory previously which caused noodle spoilage during storage. In this study, we investigated the antibacterial action of curcumin against these two bacteria by detecting membrane integrity, intracellular biomolecule leakage, and cellular morphology and assessed the practical application of curcumin in millet fresh noodle for quality and safety control through shelf life examination and sensory evaluation.

## 2 MATERIALS AND METHODS

### 2.1 Materials

Curcumin was obtained from Sigma‐Aldrich and was dissolved in ethanol (food grade, 95.5% purity; Tianjin Zhonghe Shengtai Chemical Co. Ltd.) at a concentration of 10 mM and stored in dark at −20°C before use. Wheat flour was purchased from a local market (Hebei Jinsha River Group Co. Ltd.) and millet flour (Shanxi Dongfang Liang Life Technology Co., Ltd.) was prepared by our laboratory.

### 2.2 Preparation of millet fresh noodle with CUR

The fresh noodle used in this study contained mixed powder of wheat and millet (w/w of 1:1) and distilled water. CUR was added into 100 g mixed powder to reach a concentration of 0.05% (w/w). Dough mixer (JHMZ; Dongfu Jiuheng Instrument Co. Ltd.) was used to prepare the dough. The dough was placed in sterile plastic bag and let stand for 30 min at 30°C, followed by passing through a noodle‐making machine (JMTD 168; Dongfu Jiuheng Instrument Co. Ltd.) to produce fresh noodle with a thickness of 1.0 mm and wideness of 2.0 mm. The noodle contained 1% NaCl with pH of 6.37 and water activity of 0.94. Fresh noodle without any treatment was used as blank, and the one containing equal amount of ethanol solvent without CUR was used as control. The prepared noodle samples were used directly for following measurements.

### 2.3 Total plate count measurement

Millet fresh noodle stored under 25°C was used for total plate count (TPC). At each sampling interval, 25 g of fresh noodle was diluted with 225 ml of sterile saline in a sterile bag and homogenized for 2 min using a stomacher (BagMixer 400 CC; Interscience Company). The homogenates were serially diluted by using sterile saline and plated on plate count agar (PCA, 5.0 g tryptone, 2.5 g yeast extract, 1.0 g glucose, 15 g agar dissolved in 1,000 ml distilled water). The plates were incubated at 37°C for 24 hr in an incubator (BPX‐52; Boxun Scientific Instruments Co. Ltd.). The TPC was calculated by the colony‐counting method, and data were reported as colony‐forming units (log CFU/g).

### 2.4 Quality and sensory evaluation

The color of the dough sheets (the size of 7 × 7 cm) with or without CUR treatment was measured using a chroma meter (CR 400; Konica Minolta, Inc.) with the CIE color system (L*, a* and b*).

Texture properties were measured by a texture profile analyzer (TMS‐Pilot; Food Technology Corporation). Twenty grams of millet fresh noodle (20 cm in length) was put into 500 ml of boiling water and cooked for 2 min, then the noodle was placed in cold water immediately to cool for 30 s. Measurement was carried out at 25°C ± 1°C with the disk extrusion probe and calculated based on the texture profile analysis software at the optimal test condition (deformation, 75%; detection speed, 48 mm/min; minimum initial force, 0.05 N).

Sensory evaluation of the cooked millet fresh noodle (with or without 0.05% CUR) was performed according to Li, Ma, Zhu, Guo, & Zhou (2017). An experienced tester group (six females and six males) conducted the sensory evaluation according to the 9‐point scale with “1” representing dislike extremely and “9” representing like extremely. The quantitative descriptive analysis was conducted with seven categories, including color, odor, taste, texture, hardness, stickiness, and overall acceptability. The assessment was finished within 10 min after the noodle was cooked.

### 2.5 Strain culture

The bacterial strains used in this study were isolated from the millet noodle produced in our laboratory. The strains of *B. cereus* and *E. coli* were maintained at −80°C in Luria–Bertani (LB) broth (10 g tryptone, 5 g yeast extract, 10 g NaCl dissolved in 1,000 ml distilled water) containing 30% (v/v) glycerol. Cultures of *B. cereus* and *E. coli* were thawed at room temperature, and then, 10 μl of the stock culture was inoculated into 10 ml of sterile LB, followed by enlarged propagation by inoculating 1 ml of the culture into 100 ml of sterile LB and incubating at 37°C for 24 hr (Diao et al., 2018).

### 2.6 Minimum inhibition concentration and minimum bactericide concentration measurement

Minimum inhibition concentrations (MICs) and minimum bactericide concentrations (MBCs) of CUR against *B. cereus* and *E. coli* were determined through standard twofold serial diluting microtiter method (Zhang, Liu, Wang, Jiang, & Quek, 2016). *B. cereus* and *E. coli* cells at logarithmic phase were collected by centrifugation at 3,913 *g* for 10 min at 4°C, and then, cells were washed twice with sterile saline and resuspended in wash buffer to obtain the bacterial suspension. CUR solutions (100 μl) were prepared in sterile 96‐well microplate with fresh LB broth (100 μl) to achieve the corresponding concentrations (w/v) from 0.001 to 1.0 (0.001, 0.002, 0.004, 0.008, 0.016, 0.031, 0.062, 0.125, 0.25, 0.5, and1.0) mg/ml. Prepared bacterial cells (10^6^ CFU/ml, 100 μl) were inoculated into each plate well and incubated for 24 hr at 37°C, followed by measuring OD_600 nm_ values with a microplate reader (Infinite F200 PRO; Tecan). MIC was determined as the concentration at which no visible bacterial growth was observed. Then, 100 μl of the bacterial cultures treated with ≥1 MIC CUR was spread on LB agar plates and incubated at 37°C for 24 hr. MBC was identified as the concentration at which no visible colony on agar plate was observed.

### 2.7 Bacterial growth curve measurement

The inhibitive effect of CUR on *B. cereus* and *E. coli* growth curves was evaluated. Briefly, logarithmic phase bacterial cells were harvested by centrifuge (3,913 *g*, 10 min, 4°C) and resuspended in sterile saline to obtain a final concentration of 10^6^ CFU/ml. Aliquots of 20 μl bacterial suspension were added to a 48‐well microplate containing 500 μl sterile LB broth. CUR was added to the culture to get the concentrations of 1/2MIC, 1MIC, and 2MIC, respectively. Bacterial culture containing ethanol without CUR was used as control. The growth curves were determined by measuring the OD_600 nm_ value every 2 hr for 24 hr duration by a microplate reader (Infinite F200 PRO; Tecan) (Babii et al., 2016).

### 2.8 Leakage of intracellular substances

#### 2.8.1 Measurement of extracellular macromolecules content

The content of bacterial nucleic acid in solution was monitored by measuring the absorbance at 260 nm (Cui, Zhang, Zhou, Zhao, & Lin, 2015). Briefly, bacterial cells were collected (3,913 *g* for 10 min, at 4°C), rinsed, and resuspended to the final concentration of 10^6^ CFU/ml, followed by incubating with CUR solution (0, 1MIC and 2MIC) at 37°C with shaking (150 rpm). The cell suspensions were taken at the interval of 3, 6, and 9 hr after incubation. Then, suspensions were filtered through 0.22 μm membrane and the supernatants were collected followed by measuring at 260 nm using a UV‐VIS spectrophotometer (UV2800‐A; UNICO instruments Co. Ltd.).

The measurement of protein content in solution was performed to analyze the influence of CUR on cell membrane permeability. Different concentrations of CUR suspension (0, 1MIC, and 2MIC) and bacterial cultures (10^6^ CFU/ml) were prepared. Bacterial samples were incubated with various CUR suspensions at 37°C for 3, 6, and 9 hr with shaking (150 rpm), respectively. The supernatants of cell suspensions after centrifugation (3,913 *g*, 10 min) were collected for following measurements. The leakage of protein was determined by using BCA protein quantitation kit (R23183 Yuanye Biotechnology) and measured with a microplate reader (Infinite M200 PRO; Tecan).

#### 2.8.2 Measurement of intracellular ATP concentration

As the variation of intracellular biomolecule content could be considered as an indicator of cell membrane integrity (Sanchez, Garcia, & Heredia, 2010), the intracellular ATP concentration of bacterial cells with or without CUR treatment was determined in this study. Briefly, CUR was added to bacterial suspension (10^6^ CFU/ml) to obtain the concentrations of 0, 1MIC, and 2MIC, followed by incubating at 37°C for 30 min. Cell disintegration was achieved by ultrasonic treatment (BILON‐1000CT; Bilon Instrument Co., Ltd.) followed by centrifugation (3,913 *g*, 5 min). And the collected supernatant was kept on ice until measurement. The intracellular ATP concentration was determined by using an ATP assay kit (S0027; Beyotime Bioengineering Institute) in accordance with the manufacturer's instructions.

### 2.9 Bacterial cell morphology analysis

Scanning electron microscopy (SEM) was used for cell morphology analysis. Bacterial cells were incubated with different concentrations of CUR (0, 1MIC, and 2MIC) for 6 hr at 37°C. After CUR treatment, cells were harvested by centrifugation (3,913 *g*, 10 min, at 4°C) and the pellets were rinsed thrice with sterile PBS followed by fixation in glutaraldehyde solution (2.5%) for 2 hr. The fixed sample was centrifuged, washed with PBS, and dehydrated in a series ethanol solution (25%, 50%, 75%, 95%, and 100%). The specimens were coated with gold followed by SEM (SU8020 Scanning Electron Microscope) examination.

### 2.10 Bacterial cell viability measurement

Bacterial cell viability after CUR treatment was analyzed by flow cytometry assay (Wang, Wang, & Xie, 2010). Briefly, bacterial cells were collected by centrifugation, rinsed with sterile saline, and resuspended in the saline to a concentration of 10^6^ CFU/ml. The bacterial suspension was treated with different concentrations of CUR (0, 1MIC, and 2MIC) for 6 hr at 37°C. After incubation, the samples were centrifuged (3,913 *g*, 5 min, 4°C), and the collected cells were rinsed and resuspended in phosphate buffer saline (PBS, pH 7.4). The cell suspension was mixed with propidium iodide (PI, Solarbio Technology Co., Ltd.) and stained in dark for 10 min, followed by measuring with a CytoFLEX flow cytometry (A00‐1‐1102; Beckman Coulter Inc.) immediately.

### 2.11 Statistical analysis

The data obtained in this experiment were expressed as the mean of at least three repeat measurements and standard deviation (*SD*). Data were subjected to analysis of variance (ANOVA) by using SPSS 25.0 statistics software. Significance was defined by using least significant difference (LSD) procedure and Duncan's test.

## 3 RESULTS

### 3.1 Effect of CUR on TPC of millet noodle

**Figure 1:**
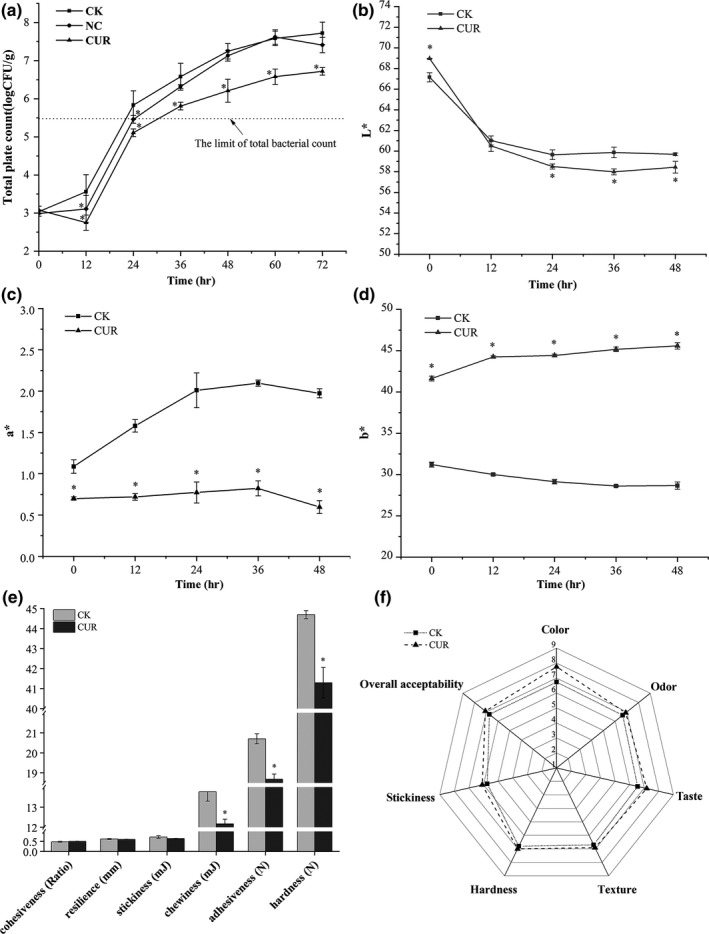
Effect of curcumin on millet fresh noodle during storage. Changes of total plate counts (a), color (b–d), texture (e), and sensory evaluation (f) in all samples. CK: without any treatment; NC: treatment with ethanol; CUR: treatment with curcumin, *p < .05 versus the control group

Total plate count of millet fresh noodle during 72 hr of storage at 25°C is presented in Figure 1a. The result showed that TPC of noodle sample with CUR treatment increased much more slowly compared with the one without CUR treatment (CK) and the one with equal amount of ethanol treatment (NC), indicating an effective antibacterial activity of CUR. During the first 24 hr storage period, TPC of millet noodle without any treatment increased from 3.04 log (CFU/g) to 5.83 log (CFU/g), reaching 5.48 log (CFU/g) at hour 22, which was the limit detection of total bacterial count in noodle products (Su et al., 2019). Although ethanol had similar inhibitory effect during the first 24 hr, the effect was not significant after that time point (*p* > .05). Noodle sample with 0.05% CUR treatment exhibited an obvious delay in bacterial growth (*p* < .05) and an extended shelf life of 30 hr. These results indicated that bacterial growth was effectively inhibited within the storage period after the addition of CUR, and the shelf life of fresh noodle was extended by 36.4%.

### 3.2 Effect of CUR on quality and sensory acceptability of millet noodle

The changes of color, texture parameters, and sensory score of the millet noodle after CUR treatment are shown in Figure 1b–f. As Figure 1b showed, noodle after CUR treatment had a higher L* value at the starting point (0 hr), while a lower L* value during the storage period of 24–48 hr (*p* < .05). The a* value decreased while b* value increased significantly (*p* < .05) after CUR treatment (Figure 1c,d). In addition, hardness, adhesiveness, and chewiness of the noodle decreased significantly (*p* < .05) after CUR treatment, while other parameters including cohesiveness, resilience, and stickiness showed no significant difference (*p* > .05) (Figure 1e). The result of sensory evaluation showed that millet noodle containing CUR had a higher score in color (*p* < .05) without undesirable taste and odor. For other characteristics such as texture, hardness, stickiness showed no significant difference (*p* > .05). Furthermore, the result of sensory evaluation showed that millet noodle with addition of CUR had a higher score in color (*p* < .05). Undesirable taste and odor were not found in the noodle containing CUR. For other characteristics such as texture, hardness and stickiness, and overall acceptability, no significant differences were discovered. Thus, the application of CUR as an additive in millet fresh noodle would not cause negative effect in product quality and sensory characteristics.

### 3.3 MICs and MBCs against the spoilage bacteria

The MICs of CUR against *B. cereus* and *E. coli* were 0.125 and 0.5 mg/ml, respectively, while the MBCs of the two bacteria were 0.5 and 1 mg/ml, respectively. CUR revealed stronger antibacterial effect and lower MIC/MBC against *B. cereus* than *E. coli*.

### 3.4 Inhibitory effect of CUR on bacterial growth curve

**Figure 2:**
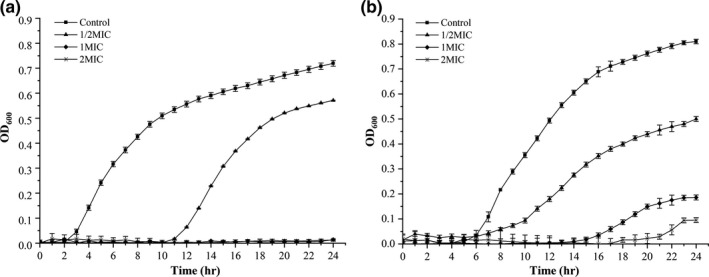
Effect of curcumin on the growth curves of *Bacillus cereus* (a) and *Escherichia coli* (b)

As shown in Figure 2, *B. cereus* and *E. coli* cells with CUR treatment (1/2MIC, 1MIC, and 2MIC) had an obvious delay in bacterial growth compared to the one without CUR treatment. The control of *B. cereus* and *E. coli* cells exhibited a S‐shape growth curve, showing a lag phase at the first 3 and 6 hr, a logarithmic growth phase till hours 10 and 15, and a stable phase after hours 14 and 18, respectively. After the treatment of 1/2MIC CUR, the growth curve of *B. cereus* and *E. coli* still exhibited a S‐shape, while the logarithmic growth phase of both bacteria was significantly suspended until after hours 12 and 10, respectively. When the concentration of CUR was increased to 1MIC and 2MIC, the growth of *B. cereus* was completely inhibited (Figure 2a). And the logarithmic growth phase of *E. coli* was delayed to hours 16 and 21, respectively (Figure 2b). These results indicated that CUR exhibited significant inhibitory effect for both bacteria in a dose–effect relationship.

### 3.5 Leakage of intracellular substances

#### 3.5.1 Changes in extracellular macromolecules content

**Figure 3:**
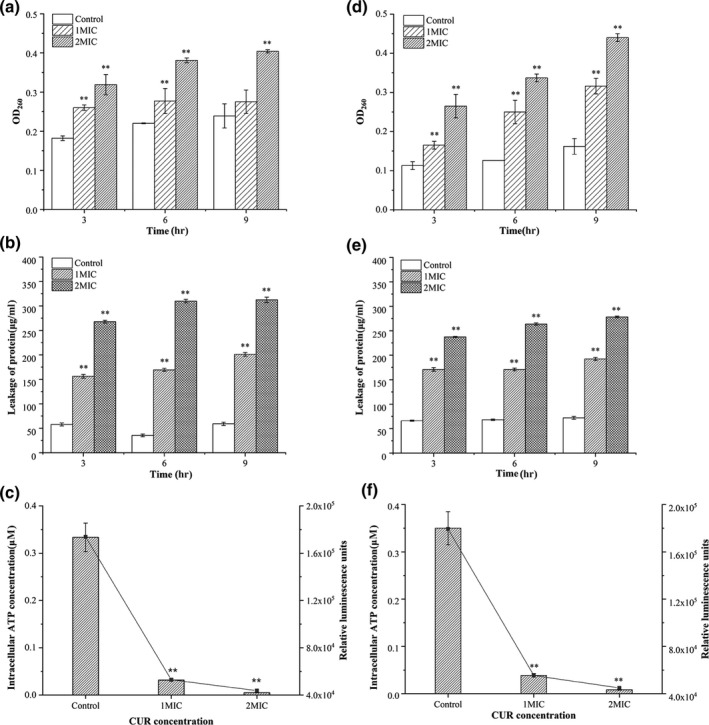
Effect of curcumin on the leakage of intracellular substances, including extracellular nucleic acid, soluble protein, and intracellular ATP of *Bacillus cereus* (a–c) and *Escherichia coli* (d–f), respectively, **p < .01 versus the control group

As shown in Figure 3a,d, there was an obvious increase in OD_260_ after 3 hr treatment of CUR at 1MIC and 2MIC for both bacteria (*p* < .05), indicating the leakage of nucleic acid from cell into extracellular environment. After 6 hr treatment, the absorbance of *B. cereus* suspensions increased by 0.057 and 0.161 (Figure 3a). And for *E. coli* suspension, the absorbance increased by 0.124 and 0.211, respectively (Figure 3d). The absorbance of *B. cereus* and *E. coli* suspensions after 9 hr CUR treatment increased in the same trend. In addition, the amounts of released nucleic acid increased with the increasing of CUR concentration.

The soluble protein content in bacterial cultures exposed to CUR was compared with that of untreated cells via BCA assay. The concentration of extracellular protein was kept at around 50 μg/ml for both bacterial cells without CUR treatment (Figure 3b,e). After 1MIC and 2MIC CUR treatment for 3 hr, the protein concentration in *B. cereus* culture increased significantly (*p* < .05) and the content was close to 156 and 268 μg/ml, respectively (Figure 3b). The protein content in *E. coli* culture after the same treatment was increased to 171 and 237 μg/ml, respectively (Figure 3e). The same trend was observed in longer hour treatment samples, and the protein concentration increased along with the increase of CUR concentration.

#### 3.5.2 Changes in intracellular ATP concentration

As shown in Figure 3c,f, the intracellular ATP concentration and relative fluorescence intensity of *B. cereus* and *E. coli* cultures were significantly reduced after treatment with different concentrations of CUR (*p* < .05). The higher the CUR concentration, the lower the intracellular ATP concentration and fluorescence intensity. After 6 hr CUR treatment at 1MIC and 2MIC, intracellular ATP concentration of *B. cereus* decreased from 0.3335 μmol/L to 0.0316 μmol/L and 0.0049 μmol/L, and the corresponding florescence intensity decreased from 1.74 × 10^5^ to 5.29 × 10^4^ and 4.38 × 10^4^, respectively (Figure 3c). For *E. coli* cells, the ATP concentration after same treatment was reduced to 0.0387 and 0.0083 μmol/L, while the florescence intensity decreased from 1.79 × 10^5^ to 5.54 × 10^4^ and 4.49 × 10^4^, respectively (Figure 3f).

### 3.6 Effect of CUR on bacterial cell morphology

**Figure 4:**
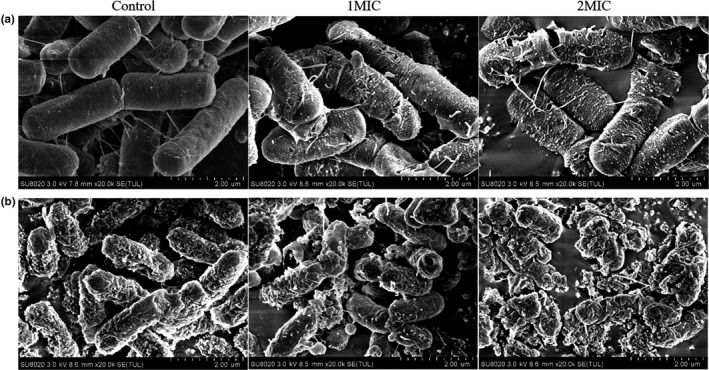
SEM images of *Bacillus cereus* (a) and *Escherichia coli* (b) cells treated with different concentrations of curcumin

The bacterial cell morphology of *B. cereus* and *E. coli* with different concentration of CUR treatments was observed by SEM. The image of *B. cereus* in the control group revealed a normal cell morphology with typical rod‐shaped and intact structure, without notable rupture pores (Figure 4a). The cells exposed to 1MIC CUR showed an obvious deformation and porous structure, compared with the smooth surface of the control. In addition, bacterial cell treated with 2MIC CUR displayed severe surface collapse and morphological destruction, resulting in larger amounts of abnormal cells and leaking of intracellular contents. Similar observations were found in *E. coli* cells after the same treatment (Figure 4b), resulting in notable aggregation of cellular debris. The degree of disintegration and cytoplasmic leakage increased with the increase of CUR concentration. These results suggested that CUR could disrupt cell membrane of both cells, causing deformation and ultimately cell death.

### 3.7 Effect of CUR on cell viability

**Figure 5:**
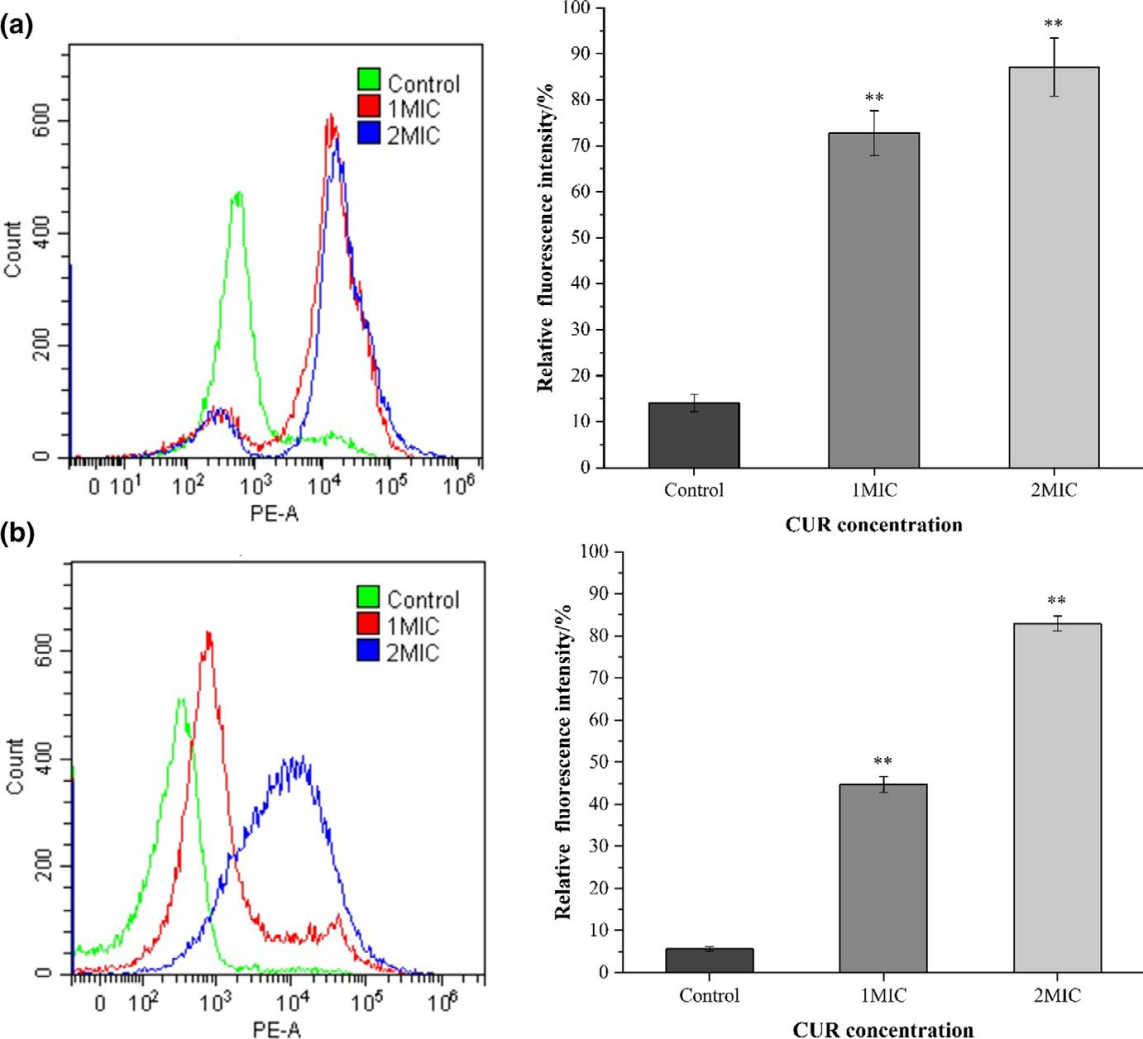
Fluorescence intensity of PI single staining of *Bacillus cereus* (a) and *Escherichia coli* (b) treated with different concentrations of curcumin, **p < .01 versus the control group

The cell viability of *B. cereus* and *E. coli* is shown in Figure 5. The highest PI signals were detected from bacterial cells after 2MIC CUR treatment. After treating with 1MIC and 2MIC CUR, the relative fluorescence intensity of *B. cereus* increased from 14.05% to 72.75% and 87.06%, respectively (Figure 5a). For *E. coli* cells after the same treatment, intensity increased to 39.06% and 77.24%, respectively, compared to the control group (Figure 5b). The fluorescence intensity increased along with the increase of CUR concentration, indicating a positive correlation between CUR concentration and membrane integrity disruption. Our results indicated that CUR could cause the malfunction of cell permeability barrier and reduce cell viability, resulting in the staining of PI with nucleic acids.

## 4 DISCUSSION

Fresh ready‐to‐eat food, such as millet fresh noodle, has been implicated in short expiration date and easy of deterioration. Effective preservation method should control the growth of spoilage microorganism while not cause unacceptable quality changes. CUR as a natural ingredient which is extracted from Curcuma longa plant has been proved to possess good inhibitory effect on food‐borne pathogen. Curcumin with a dose of 0.5% could significantly decrease the count of *L. monocytogenes* and *S. aureus* in minced meat by 2 log CFU/g and 1 log CFU/g (Sandikci Altunatmaz et al., 2016). In our study, CUR was found to prolong the shelf life of millet fresh noodle from 20 to 30 hr during storage at 25°C (Figure 1a). Changes of color (L*, a*, and b*) after CUR treatment were shown in Figure 1b–d, which was consistent with the photograph of millet fresh noodle displayed in Figure S1. Similarly, the addition of turmeric powder (2%, 4%, 6%, and 8%) was reported to decrease the L* value and increase the b* value due to its natural yellow color (Lim, Park, Ghafoor, Hwang, & Park, 2011). The texture parameters of hardness, adhesiveness, and chewiness of the noodle decreased significantly (*p* < .05) with the addition of CUR, while there was no significant difference in cohesiveness, resilience, and stickiness (Figure 1e). Sensory evaluation indicated that noodles with or without CUR treatment did not differ in taste, odor, and overall acceptability (Figure 1f). The result of sensory scores showed that the changes in color and texture property of millet fresh noodle were acceptable, and thus, application of CUR could improve the quality of millet noodle without decline in sensory quality.

The growth and reproduction of microorganisms in suitable condition (e.g., high moisture content and rich nutrition substrate) are the main cause of deterioration of millet fresh noodle (Rosyid, Karim, Adzahan, Ghazali, & Ghazali, 2011; Xu, Hall, Wolf‐Hall, & Manthey, 2008). In this study, CUR was found to inhibit the growth of *B. cereus* and *E. coli* with different susceptibility. Similarly, Sathishkumar et al. (2015) indicated that MIC of CUR dissolved in DMSO against *B. cereus* and *E. coli* was 0.2 and 0.35 mg/ml, while Gunes et al. (2013) reported that MIC of CUR dissolved in ethanol against *B. subtilis* and *E. coli* was 0.129 and 0.163 mg/ml, respectively. To further verify the inhibitory activity of CUR against the growth of *B. cereus* and *E. coli*, bacterial growth curve with CUR treatment was investigated. The results showed that growth of *B. cereus* was completely inhibited in the presence of CUR at 1MIC and 2MIC (Figure 2a), while the logarithmic phase of *E. coli* was extended at both concentrations (Figure 2b).

The changes of extracellular biomolecules in *B. cereus* and *E. coli* supernatant after CUR treatment were shown in Figure 3. There was a significant increase (*p* < .05) in OD_260_ after 3, 6, and 9 hr treatment of CUR at 1MIC and 2MIC for both bacteria (Figure 3a,d). As protein is crucial to bacterial life activities and physiological functions, the leakage of which could indicate the anomalous structure of cell membrane and lead to malfunction and cell death. Thus, the increased extracellular protein content of *B. cereus* and *E. coli* after CUR treatment (Figure 3b,e) indicated the loss of cellular membrane integrity. The intracellular ATP concentration and relative fluorescence decreased after different concentrations of CUR treatment (Figure 3c,f) which was possibly associated with leakage of intracellular ATP and depletion of the intracellular ATP pool. In general, the intracellular ATP concentration was considered as a primary factor which could provide energy for cell normal physiological activities (Gill & Holley, 2004). These results suggested that the loss of ATP might be related to the destruction of cell membrane integrity.

To confirm the inhibitory mechanism, micromorphology of *B. cereus* and *E. coli* was observed by SEM (Figure 4). The SEM images revealed an obvious shrinkage and disruption of cell membrane as well as leakage of intracellular contents for both cells. These findings were also in accordance with the changes of nucleic acid, protein, and ATP content of CUR treated bacterial cells. Propidium iodide as a fluorescent stain is commonly used to evaluate cell membrane permeability and microbial viability. For normal cells, with an intact membrane, PI is not able to penetrate into cells and intercalate the structure of nucleic acid, while for disrupted or dead cells, PI could penetrate and stain by emitting a fluorescent signal (Ueckert et al., 1995). As shown in Figure 5, the relative fluorescence intensity for both bacteria increased significantly (*p* < .05) after treating with 1MIC and 2MIC CUR which indicated that CUR decreased cell viability by damaging cell membrane. Taken together, the antimicrobial mechanism of CUR against *B. cereus* and *E. coli* was in correlation with the structure alteration of bacterial cell membrane, the resulted porous surface, and intracellular contents leakage.

## 5 CONCLUSION

The improvement in storage period and sensory acceptance of millet fresh noodle suggested that CUR, a natural product derived from plant, had the potential to be applied in noodle products as an alternative preservative. CUR showed good antibacterial effects against *B. cereus* and *E. coli* in vitro. Results of cell membrane integrity, cell viability, and microstructural changes demonstrated that CUR could disrupt bacterial membrane, causing depressions and holes, cell lysis, and ultimately cell death. However, the study on other dominant spoilage microorganisms, including fungus and yeast, and the interaction of CUR with complex components in food matrix should be further performed to better elucidate the antimicrobial effect of CUR.

## CONFLICT OF INTEREST

The authors declare no conflict of interest.

## ETHICAL APPROVAL

The experiment does not include any animal or human testing.

## References

[fsn31427-bib-0001] Anto, R. J. , George, J. , Babu, K. V. D. , Rajasekharan, K. N. , & Kuttan, R. (1996). Antimutagenic and anticarcinogenic activity of natural and synthetic curcuminoids. Mutation Research‐Genetic Toxicology, 370(2), 127–131. 10.1016/0165-1218(96)00074-2 8879271

[fsn31427-bib-0002] Asouri, M. , Ataee, R. , Ahmadi, A. A. , Amini, A. , & Moshaei, M. R. (2013). Antioxidant and free radical scavenging activities of curcumin. Asian Journal of Chemistry, 25(13), 7593–7595. 10.14233/ajchem.2013.15308

[fsn31427-bib-0003] Babii, C. , Bahrin, L. G. , Neagu, A. N. , Gostin, I. , Mihasan, M. , Birsa, L. M. , & Stefan, M. (2016). Antibacterial activity and proposed action mechanism of a new class of synthetic tricyclic flavonoids. Journal of Applied Microbiology, 120(3), 630–637. 10.1111/jam.13048 26744255

[fsn31427-bib-0004] Charles, A. L. , Huang, T. C. , Lai, P. Y. , Chen, C. C. , Lee, P. P. , & Chang, Y. H. (2007). Study of wheat flour–cassava starch composite mix and the function of cassava mucilage in Chinese noodles. Food Hydrocolloids, 21(3), 368–378. 10.1016/j.foodhyd.2006.04.008

[fsn31427-bib-0005] Chen, Z. , Schols, H. A. , & Voragen, A. G. J. (2003). The use of potato and sweet potato starches affects white salted noodle quality. Journal of Food Science, 68(9), 2630–2637. 10.1111/j.1365-2621.2003.tb05781.x

[fsn31427-bib-0006] Cui, H. , Zhang, X. , Zhou, H. , Zhao, C. , & Lin, L. (2015). Antimicrobial activity and mechanisms of *Salvia sclarea* essential oil. Botanical Studies, 56(1), 16 10.1186/s40529-015-0096-4 28510825 PMC5432889

[fsn31427-bib-0007] Diao, M. , Qi, D. , Xu, M. , Lu, Z. , Lv, F. , Bie, X. , … Zhao, H. (2018). Antibacterial activity and mechanism of monolauroyl‐galactosylglycerol against *Bacillus cereus* . Food Control, 85, 339–344. 10.1016/j.foodcont.2017.10.019

[fsn31427-bib-0008] Fei, P. , Ali, M. A. , Gong, S. , Sun, Q. , Bi, X. , Liu, S. , & Guo, L. (2018). Antimicrobial activity and mechanism of action of olive oil polyphenols extract against *Cronobacter sakazakii* . Food Control, 94, 289–294. 10.1016/j.foodcont.2018.07.022

[fsn31427-bib-0009] Fei, P. , Xu, Y. , Zhao, S. , Gong, S. , & Guo, L. (2019). Olive oil polyphenol extract inhibits vegetative cells of *Bacillus cereus* isolated from raw milk. Journal of Dairy Science, 102(5), 3894–3902. 10.3168/jds.2018-15184 30852028

[fsn31427-bib-0010] Gill, A. O. , & Holley, R. A. (2004). Mechanisms of bactericidal action of cinnamaldehyde against *Listeria monocytogenes* and of eugenol against *L. monocytogenes* and *Lactobacillus sakei* . Applied and Environmental Microbiology, 70(10), 5750–5755. 10.1128/AEM.70.10.5750 15466510 PMC522076

[fsn31427-bib-0011] Gunes, H. , Gulen, D. , Mutlu, R. , Gumus, A. , Tas, T. , & Topkaya, A. E. (2013). Antibacterial effects of curcumin. Toxicology and Industrial Health, 32(2), 246–250. 10.1177/0748233713498458 24097361

[fsn31427-bib-0012] Hou, J. , Li, Y. , Wang, Z. , Sun, G. , & Mo, H. (2017). Applicative effect of glycinin basic polypeptide in fresh wet noodles and antifungal characteristics. LWT – Food Science and Technology, 83, 267–274. 10.1016/j.lwt.2017.05.028

[fsn31427-bib-0013] Lao, C. D. , Ruffin, M. T. , Normolle, D. , Heath, D. D. , Murray, S. I. , Bailey, J. M. , … Brenner, D. E. (2006). Dose escalation of a curcuminoid formulation. BMC Complementary and Alternative Medicine, 6(1), 10 10.1186/1472-6882-6-10 16545122 PMC1434783

[fsn31427-bib-0014] Lee, N. , Kang, C. , & Kim, H. (2017). Effects of *γ*‐irradiation on the quality changes of fresh noodles prepared from wheat cultivated with N‐fertilization treatments. Food Science and Biotechnology, 26(1), 135–142. 10.1007/s10068-017-0018-1 30263520 PMC6049498

[fsn31427-bib-0015] Li, M. , Ma, M. , Zhu, K.-X. , Guo, X.-N. , & Zhou, H.-M. (2017). Delineating the physico-chemical, structural, and water characteristic changes during the deterioration of fresh noodles. Food Chemistry, 216374–216381. 10.1016/j.foodchem.2016.08.059 27596433

[fsn31427-bib-0016] Lim, H. S. , Park, S. H. , Ghafoor, K. , Hwang, S. Y. , & Park, J. (2011). Quality and antioxidant properties of bread containing turmeric (*Curcuma longa* L.) cultivated in South Korea. Food Chemistry, 124(4), 1577–1582. 10.1016/j.foodchem.2010.08.016

[fsn31427-bib-0017] Liu, J. , Hao, Y. , Wang, Z. , Ni, F. , Wang, Y. , Gong, L. , … Wang, J. (2018). Identification, quantification, and anti‐inflammatory activity of 5‐n‐alkylresorcinols from 21 different wheat varieties. Journal of Agricultural and Food Chemistry, 66(35), 9241–9247. 10.1021/acs.jafc.8b02911 30107738

[fsn31427-bib-0018] Rosyid, T. A. , Karim, O. , Adzahan, N. M. , Ghazali, F. M. , & Ghazali, F. M. (2011). Antibacterial activity of several Malaysian leaves extracts on the spoilage bacteria of yellow alkaline noodles. African Journal of Microbiology Research, 5(8), 898–904. 10.5897/AJMR10.762

[fsn31427-bib-0019] Sanchez, E. , Garcia, S. , & Heredia, N. (2010). Extracts of edible and medicinal plants damage membranes of *Vibrio cholerae* . Applied and Environmental Microbiology, 76(20), 6888–6894. 10.1128/AEM.03052-09 20802077 PMC2953017

[fsn31427-bib-0020] Sandikci Altunatmaz, S. , Yilmaz Aksu, F. , Issa, G. , Basaran Kahraman, B. , Dulger Altiner, D. , Buyukunal, S. K. (2016). Antimicrobial effects of curcumin against *L. monocytogenes*, *S. aureus*, *S. typhimurium* and *E. coli* O157:H7 pathogens in minced meat. Veterinární Medicína, 61(5), 256–262. 10.17221/8880-VETMED.

[fsn31427-bib-0021] Sathishkumar, P. , Hemalatha, S. , Arulkumar, M. , Ravikumar, R. , Yusoff, A. R. M. , Hadibarata, T. , & Palvannan, T. (2015). Curcuminoid extraction from turmeric (*Curcuma Longa* L.): Efficacy of bromine‐modified curcuminoids against food spoilage flora. Journal of Food Biochemistry, 39(3), 325–333. 10.1111/jfbc.12133

[fsn31427-bib-0022] Shoba, G. , Joy, D. , Joseph, T. , Majeed, M. , Rajendran, R. , & Srinivas, P. S. (1998). Influence of piperine on the pharmacokinetics of curcumin in animals and human volunteers. Planta Medica, 64(4), 353–356. 10.1055/s-2006-957450 9619120

[fsn31427-bib-0023] Srikaeo, K. , Mingyai, S. , & Sopade, P. A. (2011). Physicochemical properties, resistant starch content and enzymatic digestibility of unripe banana, edible canna, taro flours and their rice noodle products. International Journal of Food Science & Technology, 46(10), 2111–2117. 10.1111/j.1365-2621.2011.02724

[fsn31427-bib-0024] Su, R. , Li, T. , Fan, D. , Huang, J. , Zhao, J. , Yan, B. , … Zhang, H. (2019). The inhibition mechanism of ε‐polylysine against *Bacillus cereus* emerging in surimi gel during refrigerated storage. Journal of the Science of Food and Agriculture, 99(6), 2922–2930. 10.1002/jsfa.9505 30471133

[fsn31427-bib-0025] Ueckert, J. , Breeuwer, P. , Abee, T. , Stephens, P. , von Caron, G. N. , & ter Steeg, P. F. (1995). Flow cytometry applications in physiological study and detection of foodborne microorganisms. International Journal of Food Microbiology, 28(2), 317–326. 10.1016/0168-1605(95)00066-6 8750676

[fsn31427-bib-0026] Verma, A. H. , Kumar, T. S. S. , Madhumathi, K. , Rubaiya, Y. , Ramalingan, M. , & Doble, M. (2019). Curcumin releasing eggshell derived carbonated apatite nanocarriers for combined anti‐cancer, anti‐inflammatory and bone regenerative therapy. Journal of Nanoscience and Nanotechnology, 19(11), 6872–6880. 10.1166/jnn.2019.16640 31039839

[fsn31427-bib-0027] Wang, S.-Y. , Huang, Q.-M. , Chen, M.-S. , Lin, Y.-P. , Rao, P.-F. , Wu, Y. , & Wu, J.-H. (2016). Preparation and evaluation of a sustained-release buckwheat noodle. Journal of the Science of Food and Agriculture, 96(8), 2660–2667. 26300406 10.1002/jsfa.7383

[fsn31427-bib-0028] Wang, Q. , Wang, H. , & Xie, M. (2010). Antibacterial mechanism of soybean isoflavone on *Staphylococcus aureus* . Archives of Microbiology, 192(11), 893–898. 10.1007/s00203-010-0617-1 20734190

[fsn31427-bib-0029] Xu, Y. , Hall, C. III , Wolf‐Hall, C. , & Manthey, F. (2008). Fungistatic activity of flaxseed in potato dextrose agar and a fresh noodle system. International Journal of Food Microbiology, 121, 262–267. 10.1016/j.ijfoodmicro.2007.11.005 18077042

[fsn31427-bib-0030] Zhang, Y. , Liu, X. , Wang, Y. , Jiang, P. , & Quek, S. (2016). Antibacterial activity and mechanism of cinnamon essential oil against Escherichia coli and Staphylococcus aureus. Food Control, 59282–59289.

