# Supplementary material for: Effect of curcumin on the quality properties of millet fresh noodle and its inhibitory mechanism against the isolated spoilage bacteria
Source: Food Sci Nutr. 2020 Feb 8;8(3):1451–60. doi: 10.1002/fsn3.1427 (PMC7063345; doi:10.1002/fsn3.1427)
Supplement: Supplementary file 1 [file FSN3-8-1451-s001.docx]

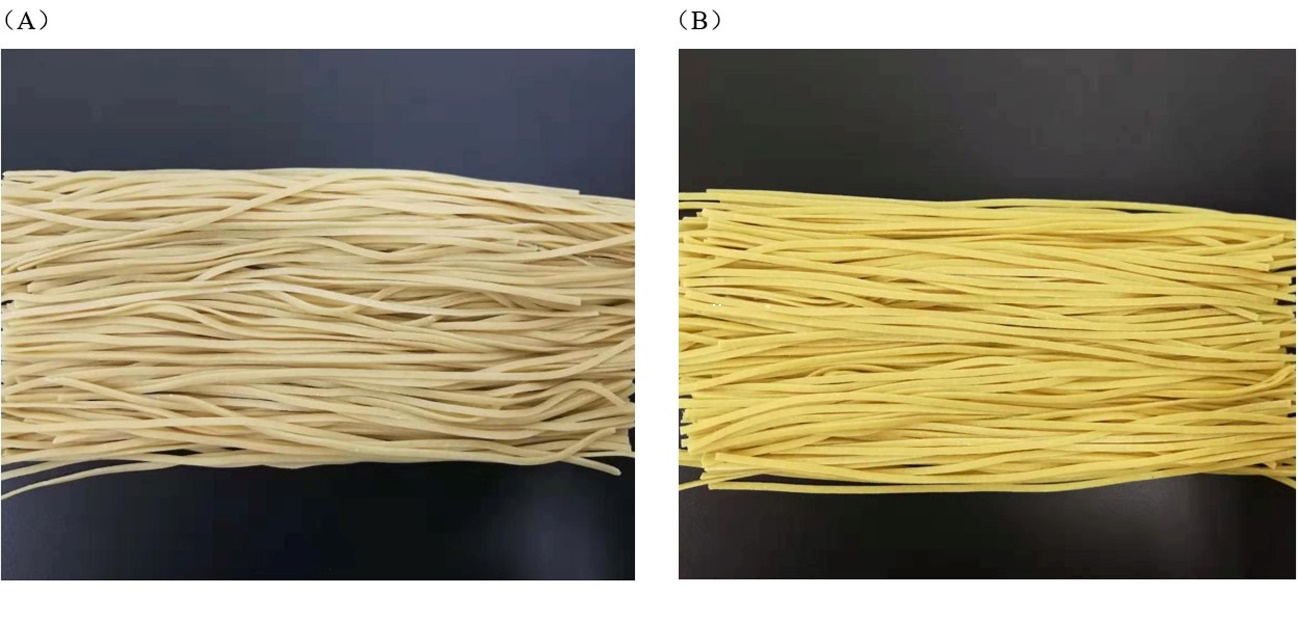


Fig. S1 Photographs of millet fresh noodle samples: A and B are noodles prepared without or with CUR treatment, respectively.
